# Supplementary material for: Actin Grips: Circular Actin-Rich Cytoskeletal Structures that Mediate the Wrapping of Polymeric Microfibers by Endothelial Cells
Source: Biomaterials. Author manuscript; Available in PMC 2016 Jun 1. (PMC4418805; doi:10.1016/j.biomaterials.2015.02.034)
Supplement: 1 [file NIHMS662697-supplement-1.docx]

1. **Introduction**

Hybrid constructs containing cells and fibrillar scaffolds are increasingly used as models of cellular interaction with extracellular matrix [1], or for various bioengineering applications [2, 3]. Because of their favorable biomimetic and biomechanical properties, cell-seeded fibrillar scaffolds are considered for vascular grafting [4], as cardiovascular patches [5, 6], or for bone reconstruction [7]. Constructs containing endothelial cells (ECs) are of particular interest, due to their relevance for vascular development and repair [6]. However, the fibrillar substrates generally used for ECs retention have diameters within either the nanometer size range [8], or larger than 50 µm [9]. This leaves a gap in our understanding of the interaction of ECs with fibers having diameters within the cell-size range (5-20 µm). Wrapping of ‘objects’ that have diameters comparable to the cells’ diameter, such as other capillaries, was proposed as a mechanism for microvascular anastomosis *in vivo* [10], but an *in vitro* model suitable for studying this process has yet to be developed. Moreover, there is a growing interest in how cell morphology and/or biomechanics concur with transcriptional and signaling pathways in regulating cellular functions such as survival, proliferation, differentiation, migration, response to stimuli, and tissue organization [11]. Thus, the attachment of cells to fibrillar scaffolds could be a method to impose shape and/or tension constrains on cells. Using it, we previously demonstrated that mesenchymal stem cells aligned by a micro-fibrillar substrate have an increased expression of cardiac differentiation-specific genes [5].

Recently we [12] and others [13] have also found that ECs incubated with polymeric scaffold microfibers (SMFs) within their own size range re-organize their actin cytoskeleton in bands oriented transversally to cylinder’s axis. In the current study, we further address the mechanisms facilitating this interaction , specifically the occurrence and maintenance of a tubular morphology in this cellular system, by focusing on the organization of F-actin as a dynamic cytoskeletal component. At the scale of interest here, ECs are organized *in vivo* as tube-shaped components of capillaries or small arterioles [14]. *In vitro*, ECs can also be induced to adopt a substrate-dependent curvature. This was shown to determine the organization of extracellular matrix secreted by endothelial-lineage cells attached to fibers with diameters in the tens/hundreds of micrometers range in a transversal, banded pattern, independent of the orientation of the cytoskeleton (actin remaining aligned longitudinally) [9]. A transversal distribution of extracellular matrix was also found in human umbilical vein ECs (HUVECs), but not in the more primitive endothelial colony forming cells (ECSFs), attached to electrospun poly-ε-caprolactone (PCL) scaffolds with fibers below 10 µm in diameter [13].

Moreover, it was reported that human brain microvascular ECs seeded on collagen-coated glass rods with diameters close to 10 µm, completely wrap around their support, unlike HUVECs [15], or human fibroblasts in similar settings [16]. This behavior made the authors assume that high-curvature substrate wrapping is a unique property of the brain capillaries [15]. This claim conflicts with other reports on HUVECs behavior, showing that these cells could fully reel around collagen struts within the same range of dimensions, and consequently modify the orientation of their actin filaments [17].

Although ultimately being a property of the F-actin bundles themselves (as shown by an elegant biomechanical model [18]), the impact of support’s curvature on the cytoskeleton-dependent cell morphology is likely to be further compounded by a material’s adhesiveness for cells. For example, PCL used in tissue engineering applications for its multiple material qualities, has poor cellular adhesiveness [19-23]. In fact, the PCL films required a chemical modification by macromolecular covalent modification [19, 20] or alkalinization [21, 22], to become an optimal culture support comparable with tissue culture polystyrene (TCPS) for HUVECs [19-21] and for human ECFCs [22], respectively. However, the behavior of EC-lineage cells on PCL scaffolds only recently started to be explored [12, 13].

Our analysis of ECs and of ECFCs engaged with SMFs prepared from non-modified PCL helps explain how these cells adapt to a geometrically unusual environment, also shedding light on their behavior in analogous situations *in vivo*. The answer resides in the profound re-organization of the actin cytoskeleton, which could biomechanically compensate for the reduced biochemical adhesiveness.

1. **Materials and Methods**

**2.1 Scaffold preparation.** Scaffolds with fibers 1-10 µm in diameter were prepared by electrospinning as previously described [24]. In brief, 8-11 % (by weight) PCL solutions were prepared by dissolution in 35°C dichloromethane via continuous stirring. After cooling to room temperature, the solution was placed in a 60-cc syringe with a 20-gauge blunt tip needle and electrospun using a high voltage DC power supply set to 23 kV, a 20 cm tip-to-substrate distance and a 15 mL/h flow rate. Electrospinning was conducted in a chamber in which the relative humidity was maintained above 90%. The electrospun fibers were deposited onto aluminum foil for 15 min; the fiber sheet was then placed in a vacuum overnight to ensure the removal of residual solvent. 18 mm×18 mm samples were cut and glued at the edges on glass coverslips for imaging analysis.

**2.2 Cells and incubations with scaffolds**. HUVECs were purchased from ScienCell (Carlsbad, CA) and cultured in ECM (ScienCell). ECFCs were purchased from Lonza (Allendale, NJ) and cultured in EGM-2 (Lonza). Cells were grown in tissue culture polystyrene T-75 flasks until they reached 80% confluence, when they were trypsinized and seeded on coverslips and PCL scaffolds for time intervals ranging from 24 h to10 days. During this time, the cells were incubated at 37°C and the medium was changed bi-weekly.

**2.3 Endothelial differentiation assay.** RNA was extracted from HUVEC grown in ECM either on TCPS, or on PCL scaffolds for 1-7 days, using RNeasy Mini Kit (Qiagen, Valencia, CA) according to the manufacturer’s protocol, tested for quality, and stored at -80ºC until use. Primers (SABiosciences/Qiagen) were diluted 1:20 with molecular-grade water, and 5 µL/well were added to 384-well plates using a Biomek^®^ FX Laboratory Automation Workstation (Beckman Coulter, Inc., Brea, CA). The plates were left to dry overnight in a sterile hood and stored covered at -20ºC until use. Quantitative real-time PCR (qRT-PCR) was performed using SYBR Green (SABiosciences/Qiagen) and a 7900HT Real-Time PCR System (Applied Biosystems/Life Technologies, Foster City, CA) operated in standard mode. All of the PCR runs contained a dissociation step. The samples were amplified in duplicate in a total volume of 5 µL. The results are expressed as the relative copy number (RCN), defined as RCN = 2^–ΔCq^ × 100, where ΔCq is the difference Cq(target) – Cq(reference) [25]. As a reference for normalization, we used the median Cq values of four endogenous controls. We analyzed the expression of a set of the following 21 genes comprising: (i) primitive markers (ABCG2, CD117/cKit, CD133/prominin, CD34, GATA4, NKX2-5, POUF5F1/Oct4); (ii) vascular function-related markers (CD31/PECAM1, CAV3/caveolin3, CDH5/VE-cadherin, CNN1/calponin, FSHR/follicle stimulating hormone receptor, KDR/VEGFR2, NES/nestin [26], NOS3/eNOS, TEK/Tie2, VWF/von Willebrand Factor, as well as ACTA2/alpha actin, ALPL/alkaline phosphatase, COL1A1/collagen I); (iii) the proliferation-associated marker MKI67; and (iv) housekeeping genes B2M/beta-2 microglobulin, CAP1, GAPDH and RPL13 (as endogenous controls). For analysis, the genes were grouped in two clusters: ten with endothelial cell-relevant functions (Cluster 1), as objectively defined by gene expression covariation in our recent study [27] (CD31, CAV3, CDH5, CNN1, FSHR, KDR, NES, NOS3, TEK, VWF), and the remaining as endothelial-irrelevant genes (Cluster 2). The data for each gene at each time point were normalized to the corresponding values of the RCN obtained in two-dimensional cultures (time = 0), then averaged to generate separate indices, and displayed as dependent on time in culture, along with the MKI67 proliferation-associated gene.

**2.4 Fluorescent staining.** At the indicated time points, cells were fixed with 3.7% paraformaldehyde (PFA) and permeablized with 0.1% Triton X-100 prior to immunocytochemistry. Anti-vinculin and anti-paxillin antibodies (Sigma-Aldrich, St. Louis, MO) were added and incubated at 4°C overnight. The following day, appropriate secondary antibodies (AlexaFluor 647-labeled, phalloidin-AlexaFluor 488, and DAPI - all from Life Technologies/Invitrogen, Grand Island, NY) were added. Actin was also visualized in TCPS- and scaffold-attached cells after transfection with a BacMam actin-GFP transduction reagent (Life Technologies/Invitrogen), according to the manufacturer’s instructions.

Additionally, coverslip-attached scaffolds containing live cells were incubated with PKH26 (Sigma-Aldrich), a hydrophobic red fluorescent label [28]. The scaffolds were mounted in Fluoromount™ (Sigma-Aldrich) and imaged using Olympus Filter FV1000 and Olympus Spectral FV1000 confocal systems (Olympus America Inc., Melville, NY). Images were viewed using the Olympus FV10-ASW software.

**2. 5 Phagocytosis assays.** Trypsinized HUVEC were incubated in suspension, under plate rotation for 30 min at 37⁰C and 5% CO_2_, with 3.5 µm diameter magnetic beads conjugated with a biotinylated mouse anti-human VEGF Receptor 2 (from MACSiBeads, Miltenyi, Auburn, CA), at a ratio of beads to cells of 20:1. The cells were then fixed, permeabilized as described, stained with AlexaFluor 488-Phalloidin and further incubated with an anti-mouse-AlexaFluor 543 antibody for detection of the beads, and with 2-(4-Amidinophenyl)-6-indolecarbamidine dihydrochloride, 4′,6-Diamidino-2-phenylindole dihydrochloride (DAPI, Sigma-Aldrich, St. Louis, MO) for visualization of nuclei.

Scaffolds containing live cells were also incubated with PKH26 (Sigma-Aldrich, St. Louis, MO), a hydrophobic red fluorescent label [28], dissolved either in the solution provided by the manufacturer to label the SFMs, or in phosphate buffer saline (PBS, pH 7.4), to induce its precipitation and to be phagocytosed by the cells.

**2.6 Confocal laser scanning, multiphoton, and live cell time-lapse microscopy.** Confocal laser scanning microscopy (CLSM) images of cell-seeded scaffolds were taken using the Olympus Spectral FV1000 confocal systems. The 488 nm krypton–argon laser was used for excitation of EGFP. Projected views of the confocal images were produced using Olympus FlowView v3.2 software. For live-cell imaging, transfected cells were seeded on 10% PCL scaffolds. Live cell imaging was performed using an Infinity3 2D array confocal scanner (Visitech Intl. Ltd.) equipped with an Olympus IX81 inverted microscope system and a 40x objective lens (N.A. 0.95). Normal cell culture medium was used during imaging. The cells were maintained at 37°C and 5% CO_2_ throughout the process. Z stack images were acquired every 5 min for 1 hour. Image z-stacks were captured using a Hamamatsu C9100 EMCCD camera using MetaMorph software (Vers.7.6.0, Molecular Devices, Sunnyvale, CA).

**2.7 Image analysis.** Digital unwrapping of the F-actin bundles from the SMFs was performed using either a Matlab program that projects the space around a cylinder on a plane [15] or, to section this space with planes parallel to cylinders’ axis, our original method [12]. In addition, to determine the properties of the scaffold inductive of cell attachment, we compared fiber diameters at cell-attachment site with overall distribution of fiber diameters in the scaffold. In brief, segmented 3D images of cell-seeded scaffolds stained with PKH26, extracted from digitized confocal images, were fitted with 1- µm long template cylinders of pre-determined diameters, as described in detail in our method paper [12]. Normalized histograms of these distributions were compared for dissimilarity by the Q-Q plot method [29]. Nuclear roundness was measured by applying Principal Component (PC) Analysis [30] to the points composing the nuclei, as the ratio of the largest PC vs. the 3^rd^ largest PC (a value of 1 corresponds to round cells). The nuclei were identified in digitized DAPI-stained three-dimensional confocal images by our original non-parametric segmentation algorithm [31].

***In vivo* implanted PCL scaffolds and Matrigel plugs.** PCL scaffolds and Matrigel plugs were implanted subcutaneously in C57-Bl6 mice as previously described [32], and retrieved after six weeks. To maintain the native cellular architecture and to image the interaction in three dimensions, minimally dissected, non-sectioned preparations were fixed immediately and stained in situ by immersion with phalloidin-Alexa 488 and counter-stained with DAPI, as described. Whole specimen fragments were mounted under optical coverslips and depth-imaged with an Olympus FV1000 Mutiphoton microscope equipped with a 25x objective lens (N.A 1.05). The images were processed with the Olympus FV10-ASW software.

**2. 8 Data analysis.** Statistics (t-Test) were performed using the Microsoft Excel 2010 capabilities. Data were presented as means ± SD and a p<0.05 was considered significant.

1. **Results**

**3.1 F-actin organization within endothelial cells attached to PCL scaffolds.** Two-dimensional PCL films are well known as poorly adhesive substrates for cell cultivation, including endothelial and smooth muscle cells [19-22]. However, HUVECs could easily attach to SMFs prepared from PCL by electrospinning in micro-fibrillar form [12, 13]. Using scanning electron microscopy (SEM), we identified cells attached directly to individual SMFs, or more seldom simultaneously to multiple fibers of smaller diameters (Fig. S1). Notably, on single SMFs, the cells displayed a very attenuated morphology, including that of nuclei, barely detectable in relief (Fig. S1). HUVECs grew vigorously on SMFs for at least 10 days, covering all available space (Fig. S2A), even when they were incubated without any pre-treatment of the scaffold, or without addition of soluble adhesiveness-enhancing factors, besides those present in the culture medium.

When grown on polystyrene tissue plates as slightly subconfluent culture, HUVECs developed the commonly linear stress fibers, as well as marginal actin-rich ruffles (Fig. 1A). However, as we [12] and others [13] have previously reported, in the cells that intimately engage individual SMFs F-actin is distributed not solely as classical stress fibers (i.e. longitudinally aligned with the scaffold’s fiber length), but also as transversal bands (Fig. 1B,C). This pattern of microfilament organization was present in various degrees (Fig. S2B), in a proportion that increased with time in culture, both in HUVECs and - in our hands, unlike in [13] - in the more primitive ECFCs (Fig. 1D), although at similar times in culture less frequently in the latter.

To understand how SMF diameter influences cell attachment, we extracted the distribution of SMFs diameters and that of the associated nuclear shape directly from 3D confocal images, specifically at HUVECs attachment sites (Fig. 2), using an original software described in [12] and [31], respectively. Counterintuitively, we found that HUVECs did not grow preferably on larger-diameter branches of the scaffold (Fig. 2A). Moreover, this analysis showed that the nuclear shape also depended on SMF diameters, becoming slightly rounder as this diameter increased (Fig. 2B). Considering that the nuclear shape follows that of the whole cell [33] as effect of a direct interaction of nuclei with F-actin filaments [34], these observations combined imply that the thinner SMFs are conducive to flatter cells, i.e. that fiber’s curvature indeed controls EC-substrate interaction as suggested [15, 18], for reasons explored below detailed below.

**3.2 Circularity of F-actin distribution within scaffold-wrapping ECs**. We further analyzed the three-dimensional distribution of F-actin microfilaments in scaffold-wrapping cells, to determine the extent of circumferential coverage of the SMFs with ECs, a property never quantified before. In cells incubated for longer time (10 days) with SMFs, we documented by digital unwrapping of confocal images a fully circumferential continuity of the majority of F-actin bands (Fig. 3A). This pattern corresponded to three-dimensional rings that occasionally crossed the nuclei (Fig. 3Ac). We could also find within the scaffold-wrapping ECs at earlier stages of cell attachment (1-2 days in culture) pools of loosely but circularly organized F-actin filaments (Fig. 3Ba,b), by digitally sectioning with cylindrical planes the space around SMFs, followed by their 2D projection (‘layered unwrapping’) (Fig. 3c,d).

In certain instances however, even in long-term cultures we could see interlaced fragments of F-actin filaments in 3-to-2 dimensional projections (Fig. 3Ac), or in optical sections (Fig. 4, arrowheads). Often these filament bundles were oriented obliquely with respect to the long axis of the SMF, and did not fully surround the cylinder, for which reason we previously named them ‘concave actin bundles’ (CAVs) [12]. The cytoplasm itself of these cells still fully wrapped the supporting SMF, as inferred from the co-existence in the same cell of complete F-actin rings (Fig. 3Ac and 4, arrows). We assume these oblique CAVs represent stages in rings’ assembly, a process which may start with longitudinally oriented stress fibers, that progressively bend and at the same time reorient in a transversal position, as proposed by a theoretic biomechanical model [18].

**3.3 Role of F-actin rings in ‘gripping’ the micro-fibrillar support.** Direct confocal optical sectioning also revealed that HUVECs engaged with the scaffold for long intervals displayed their transversally-banded F-actin more often and/or more intense at the cell’s extremities (Fig. 5A). The non-marginal (internal) microfilaments were generally thinner F-actin structures, which sometimes had a heavily beaded aspect, occasionally with the conspicuous oblique pattern (e.g. Fig. 5A, C). In isolated cells, F-actin located at cell’s ends occasionally appeared undulated (Fig. 5B), reminiscent of lamellipodial actin ruffles that we described at the leading edges of migrating ECs [35]. Therefore intriguingly, the cells’ attachment to SMFs could still be compatible with some form of cell motility along the fibers in a sleeve-sliding mode, at least in sparse-seeding conditions.

Ring-containing fiber-wrapping cells usually displayed an elongated and attenuated morphology, including that of the nuclei (Fig. 5B), in concordance with the SEM images (Fig. S1). In line with the known biomechanical coupling between the cytoskeleton and nucleus [36], this could be the result of a pressure exerted over the nuclei by (or via the cytoplasm with the contribution of) these F-actin rings (Fig. 2Ac).

We further tested the hypothesis that the circularly-organized actin microfilaments could indeed play a role in stabilizing the attachment ECs to their cylindrical substrates. This possibility was suggested by several lines of evidence (expanded below), including their presence in all partitions of the cytoplasm of single cells that separately wrapped different intersecting SMFs (Fig. 5C).

To estimate the tightness of cells’ attachment to SMFs, we incubated in different experiments live HUVECs (pre-seeded on scaffolds for 10 days), with the hydrophobic fluorescent label PKH26. We found that PKH26 strongly labeled the SMFs in regions not occupied by cells (Fig. 5D-F), and very faintly - if at all (Fig. 5F) - the cellular plasma membranes [28]. Notably, the transition between stained and unstained portions on SMFs were very sharp, and always limited by nearby F-actin rings (Fig. 5D-F). This let us assume that the F-actin rings created an effective barrier for lateral diffusion of the dye in the space occupied by SMF-attached cells (compare Fig. 5E with 5F). The tight contact between the cells and fibers at the rings’ level, which thus ‘gripped’ the scaffold, prevented the diffusion of the soluble dye underneath the cells. However, SMFs were not protected from the dye when the cells did not wrap the fiber and therefore did not form rings (Fig. 5E-F, arrowheads). To indicate their putative role in cell-fiber interaction, we suggest to name these SMF-induced cytoskeletal rings as ‘actin grips’ (AGs).

**3.4. Relationship between AGs formation and cell’s phagocytic activity.** ECs belong, together with the professional macrophages, to the ‘reticulo-endothelial system’ [37], both cell types and other epithelial cells being capable of consistent phagocytosis [38]. Thus, we expected that HUVECs likewise engage in a phagocytic activity triggered by their substrate, when attached to SMFs. In principle this could explain the wrapping of SMFs by these cells as a failed attempt to engulf the PCL fibers, or a ‘frustrated phagocytosis’. To study this mechanism, we first performed a direct test of phagocytosis, where beads of ~4 µm, a size comparable with the preferred fiber diameters at the attachment sites (Fig. 2A), covered with an anti-VEGF receptor 2 (a molecule abundantly present on ECs [39]) antibody, were incubated in suspension with HUVECs (Fig. S3A). We found that indeed these beads were taken up in F-actin limited, U-shaped phagocytic ‘cups’, and internalized into fully closed vesicles (Fig. S3B, C). However, the F-actin layer deployed around these *bona-fide* phagocytic particles had a very different texture, being much more uniform in thickness and in granularity, compared to AGs.

Next we studied the uptake by scaffold-attached HUVECs of a smaller particulate phagocytic marker, prepared from the same fluorescent label PKH26 (suspended in a more hydrophilic buffer, to induce its aggregation [28]), as routinely used as a phagocytosis assay. We found that in SMF-attached HUVECs the PKH26 particles accumulated within compartments laterally separated by AGs, without any detectable F-actin at the fiber-cell interface (Fig. S3D,E). If AGs formation represented a similar response of the cells to internalized objects, they should have been distributed as a uniform layer of F-actin continuously distributed along the SMFs, which was not the case.

We also reasoned that if the organization of F-actin in SMF-attached cells was simply the result of a frustrated phagocytosis, then AGs should readily appear during SMFs interaction with professional phagocytes and/or ECs *in vivo*, a situation occurring during ‘foreign body reaction’ [40]. In reality, AGs were completely absent in the macrophages and macrophage-derived giant cells attached to PCL scaffolds that we retrieved from mice after subcutaneous implantation (Fig. S4A). Conversely, in neovascularization-inducing hydrogel (Matrigel) plugs subcutaneously implanted in mice [32], we did find in the developing capillary ECs, although very seldom, F-actin bands with a transversal distribution, in a pattern reminiscent of, but different from, that of AGs (Fig. S4B).

**3.5 Relationship of AGs with focal adhesions and intermediate filaments.** Since the stress fibers are routinely associated with focal adhesions (FAs) at their plasma membrane attachment site to substrate [41], we analyzed in SMFs-attached cells the presence and localization of the FA components paxillin and vinculin. While these proteins were both readily detectable at the ends of stress fibers in cells adherent to a flat tissue culture surface (e.g. Fig. S5A for paxillin), as well as at the extremities of actin microfilaments distributed longitudinally on scaffolds, both of these FA molecules were absent in well-developed, fully assembled AGs (Fig. 6A-D). Absence of AG-associated FAs was consistent with the morphological observation that the circular F-actin bundles could be organized as relatively thick, continuous bands placed within the cytoplasm at a distance from the plasma membrane in contact with the SMF (Fig. 3B).

Furthermore, in flat-surface attached cells the stress fibers are often reinforced by co-localization with intermediate filaments [42] (e.g. vimentin-containing intermediate filaments, Fig. S5B). However, in the scaffold-attached HUVECs, vimentin-positive intermediate filaments were absent from the cytoplasm regions occupied by AGs (Fig. 6E,F). At the same time alpha-actinin, a standard component of contractile F-actin microfilaments [43], was readily detectable in AGs by immunocytochemistry (Fig. 6G).

Fig. 6G also illustrates a key finding of this study, that AGs occurred only (although not always) in cells attached to fibers with a diameter comparable to cells’ size, implying cylindrical wrapping. HUVEC engaging fibers with larger diameters had most of their F-actin organized in stress fibers and/or peripheral bundles (Fig. 6G), in a fashion similar to those attached *in vitro* to a flat surface (e.g., Fig. 1A), or as part of the intima of larger diameter blood vessels *in vivo*. Thus, substrate’s curvature is a necessary condition [18], but not sufficient to explain the formation of AGs, which in addition seem to require a full fiber wrapping that generates a cylindrical cell shape.

**3.6 Dynamics of F-actin-containing structures in SMFs-attached cells.** In fully differentiated ECs that intimately wrap the SMFs in culture, AGs represented the majority of F-actin (Fig. S2A). However, AGs may co-exist with longitudinally-oriented F-actin in same cells, in adjacent cells located on the same fibers, or on fibers of similar diameter (Fig. S2B). This suggests that either the mechanisms of AGs formation is more complex than a simple reaction to curvature as proposed [15, 18], or that it indicates a slow progressive reorganization of F-actin from one assembly form into another. A dynamic AGs formation and disassembly would be also compatible with a migratory polarization of SMF-wrapping cells, as suggested by Fig. 5B.

For this reason, we directly investigated the dynamics of the F-actin based cytoskeletal structures in live cells. To this end, we transfected HUVECs attached to culture dishes with a GFP-actin expressing vector, and then transferred them on PCL scaffolds. As expected, in 2-dimensional cultures GFP-labeled actin monomers became incorporated into *bona-fide* stress fibers and in marginal ruffles (Fig. S5C). We then imaged live individual AG-containing cells by time-lapse fluorescence microscopy. We focused on an AG located centrally within a scaffold-attached cell, which slowly disintegrated over a one-hour time span, while other actin-dense structures simultaneously developed at cell margins (Fig. 7A). Because the cell was placed at the intersection of three fibers, the system was mechanically unstable, thus permitting observation of the consequences of an AG’s dissolution. We analyzed time-dependently the pixel intensity in selected regions of the cell (Fig. 7B), an approach that confirmed that the F-actin patch seen in optical cross-section in the central upper side of the SMF, as well as the one underneath it, belonged to the same AG (as the GFP-actin fluorescence intensity decreased perfectly in parallel). Coincidently, F-actin intensity followed a different, although temporally coordinated kinetics in other parts of the cell (encircled, Fig. 7B). Simultaneously, the cell’s length became progressively reduced on the main attachment fiber, while extending on the perpendicular direction (Fig. 7C). These geometrical changes were in accordance with a model of SMFs constriction by AGs, and of loss of gripping power coincident with their disintegration, letting other tensions within the cell to be manifested.

**3.7 Relationship between AGs formation and differentiation status of HUVECs.** The observation that the frequency of AGs increased with time in culture, both in HUVECs and in ECFCs, raised the question whether this property was cell differentiation-dependent. AGs-induced cell flattening by itself, reflected in a corresponding nuclear deformation, was expected to impact on chromatin structure and thus on gene expression, thus modulating the differentiation status [34]. These considerations prompted us to analyze the time-dependent expression of endothelial-specific genes in HUVECs cultivated on scaffolds, as compared to those maintained in regular 2D culture. We found that the genes for a set of vascular function-related markers [27], that included VEGF receptor 2 (KDR), Tie-2, VE-cadherin (CDH5), eNOS (NOS3), von Willebrand factor, nestin, CD34, CD31(PECAM), c-Kit as well as alpha actin, were collectively expressed in the scaffold-wrapping cells at levels comparable to or higher than those in cells on flat surfaces (Fig. 8). At the same time, the transcriptional activity of a panel of endothelium-irrelevant genes (see ref. [27] and Methods), along with that of the proliferation marker MKI67 were reduced (Fig. 8). Combined, this multiplexed transcriptional profiling was indicative of a comparable or even more differentiated endothelial phenotype during SMFs cultivation, than in comparably subconfluent cells, maintained in two-dimensional cultures.

1. **Discussion**

The main finding of this study is that ECs have a phenotype-specific ability to fully wrap supports with diameters comparable to those of capillaries, even when the adhesiveness is intrinsically poor, such as for PCL. This cylindrical morphology was accompanied by the progressive reorganization of the actin-rich filaments in a ring pattern. For their putative role in cell interaction with SMFs and/or stabilization of their tubular shape, we named these structures ‘actin grips’ (AGs; the simpler term ‘actin rings’ is already in use to define the peripheral actin bands present in epithelial cells [44], or in the cytokinetic furrows [45]).

To our knowledge, although a distribution of F-actin transversal to the cell’s cylindrical support has been reported before, the whole fiber-wrapping behavior involving just one cell, was not observed in other cell types besides ECs [15, 17]. For example, when fibroblasts were seeded on cylinders with a small curvature, they oriented longitudinally to avoid a sharp bending [16]. An influence of substrate curvature on morphology and orientation and F-actin organization within ECs adherent to collagen-coated glass rods with diameters varying from 10 to 500 µm was recently reported [15]. In this study, human brain microvascular endothelial cells readily wrapped the thinnest rods, and oriented a fraction of their actin microfilaments across the cylindrical axis. However, in this report HUVECs were said to resist bending and/or to change their shape, following the substrate curvature similarly to fibroblasts. HUVECs on rods covered with collagen of all diameters tested in this study systematically took an elongated morphology, and aligned with their long axis *parallel* to that of the cylindrical support, along with that of the majority of their actin [15]. In contrast, as we [12] and others [13] have previously showed, HUVECs incubated with PCL scaffolds could organize their F-actin in transversal bands. This seemingly contradictory behavior of HUVECs in geometrically comparable conditions highlights the importance of direct molecular adhesive interaction between the cells and their environment. Our results indicate that when the adhesive forces are suboptimal, ECs could still mount an engagement with a cylindrical substrate, mostly relying on biomechanical cues, i.e. by stabilizing their natural bending propensity with secondary cytoskeletal reinforcements.

In the current study we demonstrated that HUVECs on PCL fibers with diameters in the 5-15 µm range not only organize their actin cytoskeleton in a conspicuous ring-like pattern with predilection for localization at cells edges, but also efficiently wrap their cylindrical supports, thus ensuring a firm holding of the substrate. It would be surprising if such a sophisticated biomechanical mechanism existed only in *in vitro*, and did not have an *in vivo* counterpart. This could happen for instance when ECs need to wrap around a cylindrical substrate, during wrapping-and-tapping anastomosis [10]. In this case, a too strong adhesiveness would be counterproductive, because the wrapping cell must quickly disengage to slide laterally on top of the other cell, to expose their common lumens for fusion [10]. A migration while attached to SMFs was actually supported by one of our observations (Fig. 4B), indicative of migratory polarization in a fully wrapped cell.

The possibility of dynamic engagement with a cylindrical support may be facilitated by the likely *inverse* polarization status of the attracted cells: in a capillary, each EC is polarized with the apical side facing the fluid-filled lumen, and the basal one oriented towards the external side of the empty cylinder, where it is attached to the extracellular matrix-rich basal lamina. Others [46] and we [47] have shown that the morphological integration of the newly-formed capillaries with the immediate environment involves the interaction with the inner surface of a pre-existent cylindrical ‘tunnel’, as well as the synthesis and deposition of new, cell-derived basal lamina material [48]. For this reason, the attachment of ECs onto, and wrapping around, a solid SMFs is inverse to the normal topological configuration. This may explain a weaker attachment and/or faster detachment, consistent with the absence of harder to disassemble focal adhesions.

**4.1 Identity of AGs compared to other F-actin rich structures.** Due to their unusual characteristics, we compared AGs with other F-actin-containing cellular structures. First, mature AGs do not seem to be stress fibers in the traditional sense, even if they might derive from those by re-orientation. Stress fibers are conspicuous cytoskeletal structures in culture, but *in vivo* are detectable primarily in shear-stressed ECs [49], and in general in tensed cells [50]. Second, in our system, a role for AGs in direct (biochemical) adhesion of ECs to SMFs is unlikely, because we could not co-localize with the AGs two FA-specific molecules, paxillin and vinculin, which were readily detectable at the extremities of actin filaments oriented alongside the scaffold. Furthermore, time-lapse microscopy observations on live cells suggest that AGs are more dynamic structures than the classical stress fibers. They also indicate that the actin monomers from a given AG could be exchanged with other F-actin containing structures within the same cell, as part of a tightly-controlled actin homeostasis [51], leading to complex forces within cells to be exerted on the scaffold (in our case, a contraction along the SMF). Consequently, our findings suggested that AGs may contribute to the maintenance of an *elongated* endothelial morphology on the fibrillar support, even in the absence of a FA-mediated adhesion, and that their dissolution may lead to modifications of cell shape.

Combined with their curvature, this role of AGs in cell shape control presumes a close interaction with the plasma membrane, a property of *bent* septin-containing actin bundles [45]. However, at individual fiber level, stress fibers are in general *linear* structures organized by fascin, while circularity in F-actin fibers is controlled by septins, recently identified to play a key role in organization of epithelial monolayers [52], in the formation of axon’s F-actin rich transversal ‘stripes’ [53], or of the actin rings at furrows during cytokinesis [45]. However, we did not find a relationship between AGs frequency with any nuclear feature involved in cell division, nor a preferential placement of AGs at mid-cell position, as would be required for a role in cytokinesis. Instead, AGs could be more similar to dorsal stress fibers [54], to the ‘actin arcs’ present in lamellipodia and/or ruffles of migrating cells [55], or to the short-lived ‘circular dorsal ruffles’ induced by stimulation with growth factors [56], or by exposure to biaxial mechanical stretch [57]. In general, actin ruffles can be described as waves of concentrated F-actin, initiated at the cell periphery (usually the leading edge) and progressing backwards toward the cell’s upper surface as the cell body advances forward [35]. In our case, AGs were localized mostly in the median (sometimes apparently crossing the nucleus) and in the basal portion of the cell body, coincident with a flat cell morphology, also reflected in the flatness of nuclei.

**4.2. Possible roles of AGs *in vitro*.** AGs seem to represent a unique cytoskeletal organization specifically adapted to the wrapping interaction of endothelial-lineage cells with solid, elongated objects with diameters comparable with that of cell’s own size. An implication of our findings is that fiber wrapping may largely supply or even replace cells’ need for direct adherence, required for survival [58]. In fact, we very seldom observed characteristically fragmented nuclei in these cultures (data not shown). Instead, we witnessed a robust cell proliferation and covering of the PCL fibers, expanding from the initial patches to large surfaces after two weeks in culture, while flat surfaces covered with a PCL film showed poor cell-supporting (both attachment and survival) properties. This is because FAs provide the cells with a necessary anchor for attachment, and activate a pro-survival signaling program, while in their absence the cells enter a form of apoptosis called ‘anoikis’ [58]. Similar to our observation, mesenchymal stem cells cultivated on a 3D fibrillar scaffold of much larger diameter (more than 50 μm) also showed an enhanced survival, mediated by self-secreted laminin, although no wrapping and/or F-actin rings were observed in that case [59].

Here we demonstrate that this microfilament-based cytoskeletal structure is endothelial differentiation-dependent, because it is better represented in HUVEC than in the more primitive, cord-blood derived ECFCs, and their formation in scaffold attached HUVEC parallels the expression of endothelial-specific genes in these cells. In support of the differentiation dependence of AGs formation, a previous report indicates that ECFCs were unable to re-orient their actin transversally to the fiber’s cylinder, probably because those cells were prepared in the laboratory [13]. Our ECSFs were commercial, and thus more advanced in endothelial differentiation after their *in vitro* cultivation.

Due to the natural variation in a vessel’s size, ECs have shapes ranging from almost flat to cylindrical. However, most of the work addressing the properties of ECs used 2D *in vitro* models. Physiological implications of a cylindrical morphology of ECs have mostly been considered in gel-embedded cellular models. Still, the actual presence in these models of a true endothelial differentiation, including the formation of a patent (empty) capillary lumen, was often overlooked. This is due in part to the difficulty to induce and maintain the tubular shape in a controlled manner. Thus, our scaffold-based method could provide a simple and efficient method of ECs shape manipulation.

**4.3. *In vivo* relevance of AGs.** Besides the cardiovascular system [60], a cylindrical cell shape plays a preeminent role in the behavior of other cell types, particularly during developmental morphogenesis [61, 62]. We have previously investigated the acquisition of a tubular shape by endothelial progenitor cells and/or monocyte-macrophages during cell colonization of cylindrical tunnels *in vivo* [47]. This process is instrumental during sprouting-independent, progenitor cell-driven neovascularization [63]. In these cases, the tunnel diameter is also commensurate with that of colonizing cells, because the tunnels are formed by the proteolytic activity of matrix-penetrating cells of comparable size [64].

Indications of AGs presence in ECs *in vivo* is scarce at this time. This might be due to that fact that AGs are specialized structures, needed only when ECs wrap another object. This situation is not commonly encountered in normal capillaries, where constriction produced by AGs could actually pose a risk for occlusion. If so, it would be of substantial translational interest to explore the possible pathological AGs formation in several capillary obstructive conditions, currently attributed to the constrictive activity of pericytes [65]. However, AGs formation could be critical during the physiological fusion of two capillaries during their anastomosis by the proposed ‘wrapping-and-tapping’ mechanism, where one EC was suggested to wrap around, and then to slide long another capillary [10]. If so, a deficient anastomosis due to inability to engage strongly enough the target microvessels, or to maintain the wrapping (e.g. due to a reactive oxygen-species induced actin polymerization perturbation in ECs as we have previously shown [35, 66]), may occur in the pro-oxidant conditions such as diabetes [67], leading to the microvascular rarefication and/or to the perfusion deficits characteristic of this disease [68].

**Conclusions**

Here we studied the ring-like organization of actin-rich cytoskeletal bundles in mature ECs, during their full wrapping of cylindrical objects with diameters comparable with that of capillaries. We called these cytoskeletal rings ‘actin grips’ (AGs), due to their role in stabilizing the tubular shape of the cells attached to a poorly adhesive fibrillar material. We found AGs to be dynamical structures, apparently compatible with cell sliding in this fully wrapped state around the scaffold. The finding is surprising because it demonstrates a case of abundant F-actin bundles organization in a circular fashion with a constrictive capacity as well, a function reserved in ECs only to cytokinesis furrows, and to the peri-vascular contractile cells (namely, pericytes). Besides their possible roles during specialized activities in capillaries *in vivo*, which remains to be explored, AGs may also have multiple applications in tissue engineering, such as for stabilization of ECs interaction with the scaffolds used as cell carriers, for immuno-isolation of fibrillar scaffolds in order to mitigate their foreign body reaction and post-implantation immune responses, or for controlling ECs differentiation via substrate-driven gene expression.

**Acknowledgements**

Microscopy was performed in the Campus Microscopy and Imaging Facility of the Ohio State University, and qRT-PCR was performed in Nucleic Acids Shared Resource, Comprehensive Cancer Center of the Ohio State University. SEM imaging was performed at the Department of Materials Sciences and Engineering, College of Engineering, Ohio State University.

This work was supported by NIH Grants R01 HL-096524 and RC2 AG-036559 to NIM.
