# Supplementary material for: Actin Grips: Circular Actin-Rich Cytoskeletal Structures that Mediate the Wrapping of Polymeric Microfibers by Endothelial Cells
Source: Biomaterials. Author manuscript; Available in PMC 2016 Jun 1. (PMC4418805; doi:10.1016/j.biomaterials.2015.02.034)
Supplement: 2 — Figure S1. Scanning electron microscopy (SEM) of a PCL scaffold and of attached ECs. The image shows that the scaffold-attached ECFCs could engage either a single larger fiber (double-arrow), or several smaller fibers (arrows). In the first instance the cells became very attenuated (stars), including their nuclei, still visible in relief (arrowheads). Figure S2. Extent of PCL scaffold covering with HUVECs. A. SMFs covering after 10 days of incubation; note that almost all fibers were wrapped by AGs-containing cells. B. Co-existence in cells attached to SMFs of comparable diameter of stress fiber-like actin filaments placed longitudinally vs. the scaffold (arrows), with transversally oriented AGs (arrowheads). A, B, overlay of DIC and fluorescence microscopy; two-dimensional projections of confocal z-stacks (green = F-actin, blue = DAPI). Scale bars: A: 50 μm; B: 20 μm. Figure S3. Relationship between AGs and phagocytic activity in HUVECs. A–C. Bona fide phagocytosis in HUVEC of antibody-covered polystyrene beads, providing a positive control for cortical actin organization around phagosomes in this cell type (B, arrowheads). The images represent phase contrast and fluorescence microscopy of HUVECs incubated with beads in suspension, followed by fixation, permeabilization, and staining. C. Higher magnification of B, showing the formation of an U-shaped ‘actin cup’ (arrow), characteristic for phagocytic internalization, around a more superficial bead in the process of engulfment. D. Optical sectioning through two SMFs-attached cells (F-actin, green) showing internalized PKH26 particles (arrowhead, red), in spaces limited by AGs (arrows). No F-actin organization is detectable in contact with the SMFs within these regions, arguing that AGs are not directly involved in phagocytosis. E. AGs (arrows) alternating with three phagosomes (arrowheads) within a fiber-attached cell. Note that AGs in this cell contained fewer microfilaments, and displayed a conspicuous beaded appe [file NIHMS662697-supplement-2.pptx]

## Slide 1
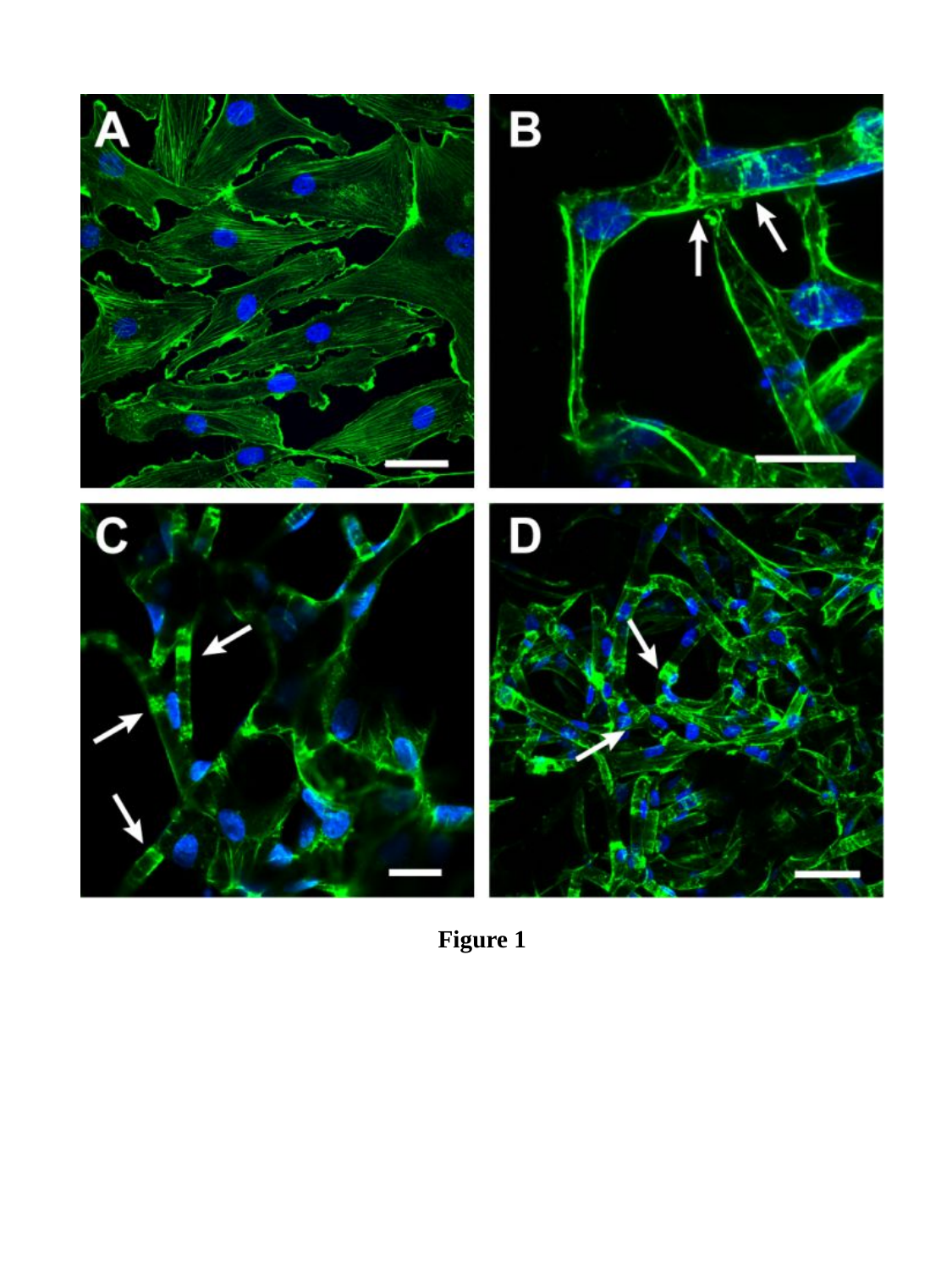

Figure 1

## Slide 2
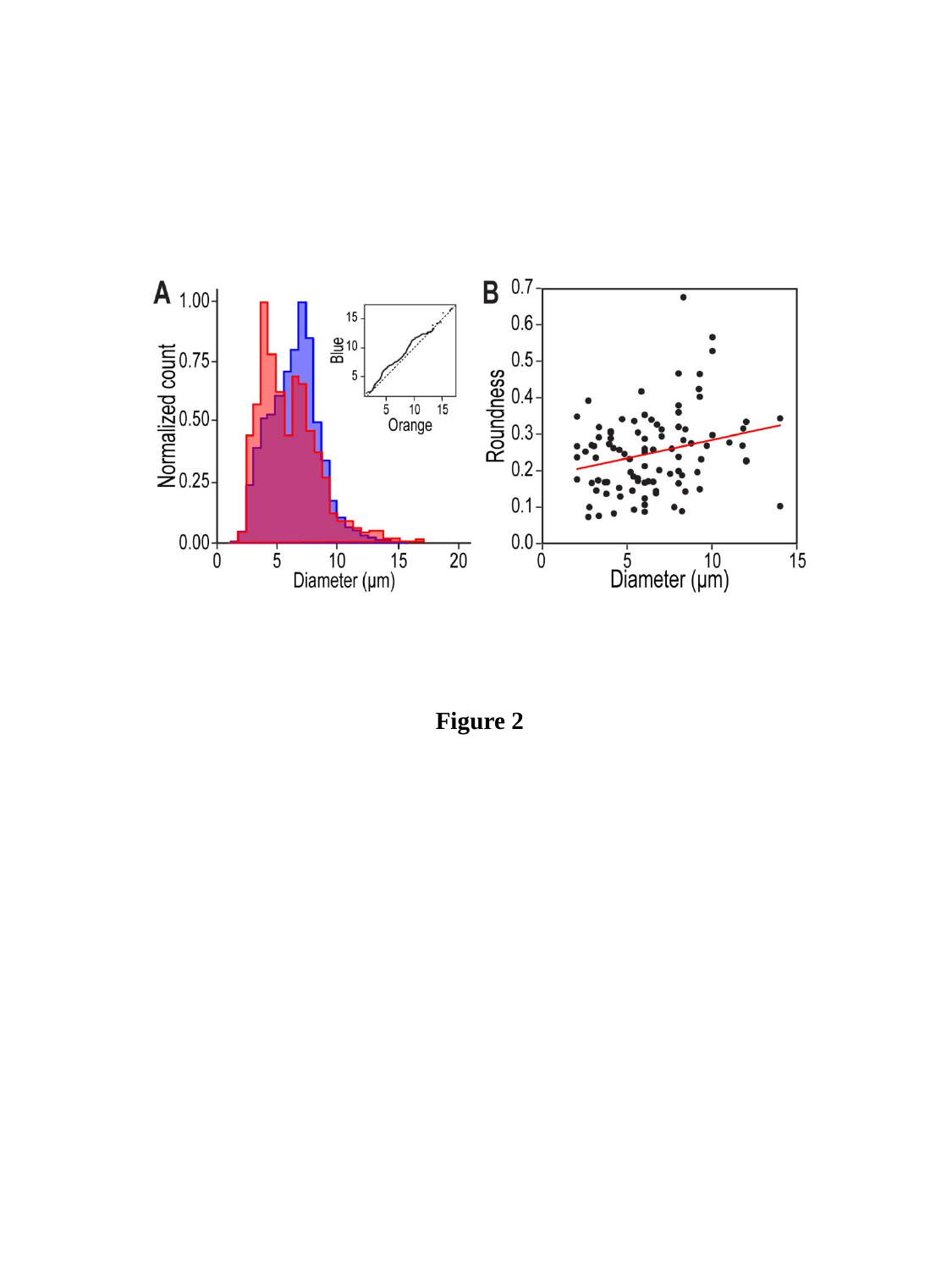

Figure 2

## Slide 3
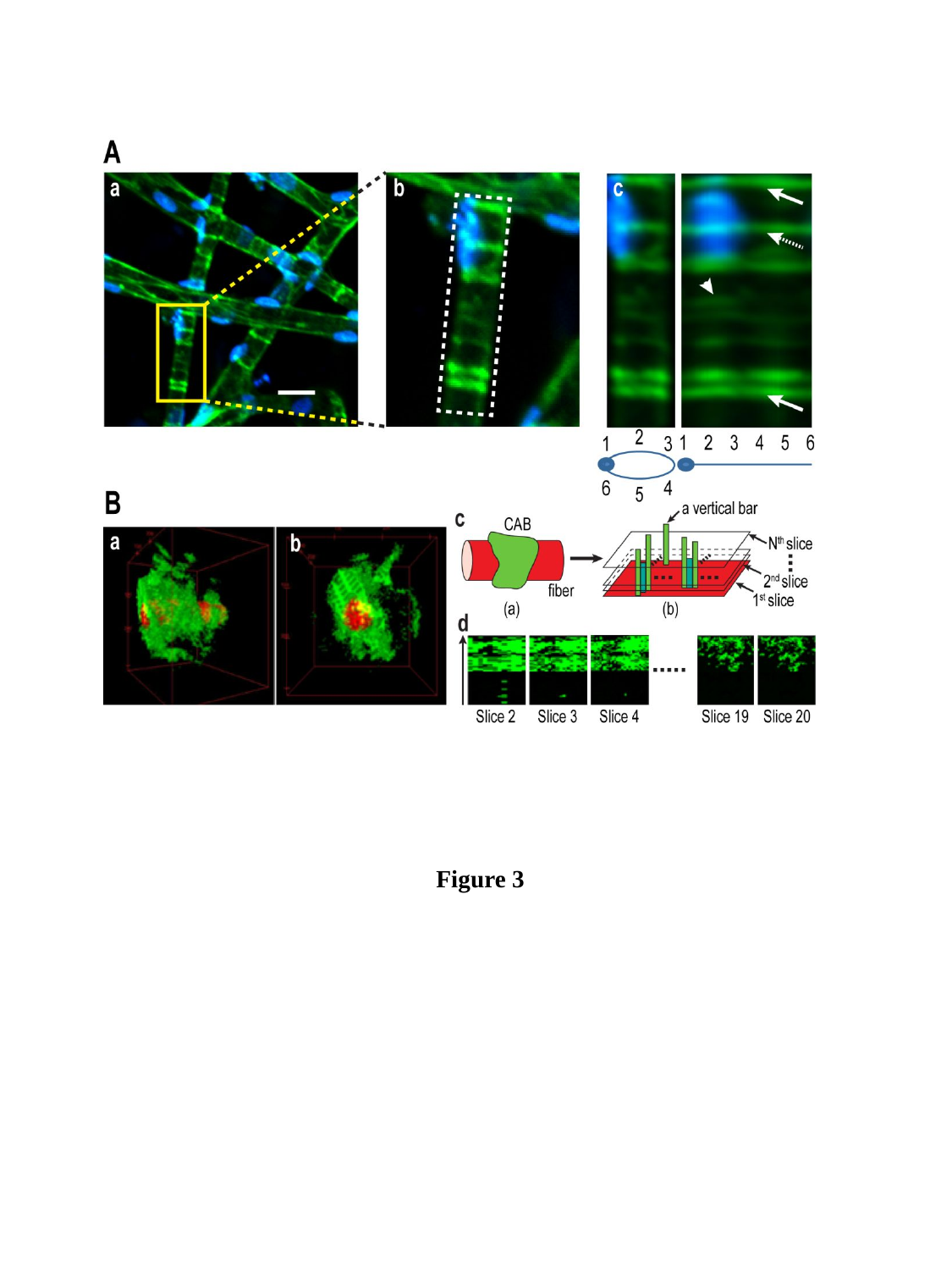

Figure 3

## Slide 4
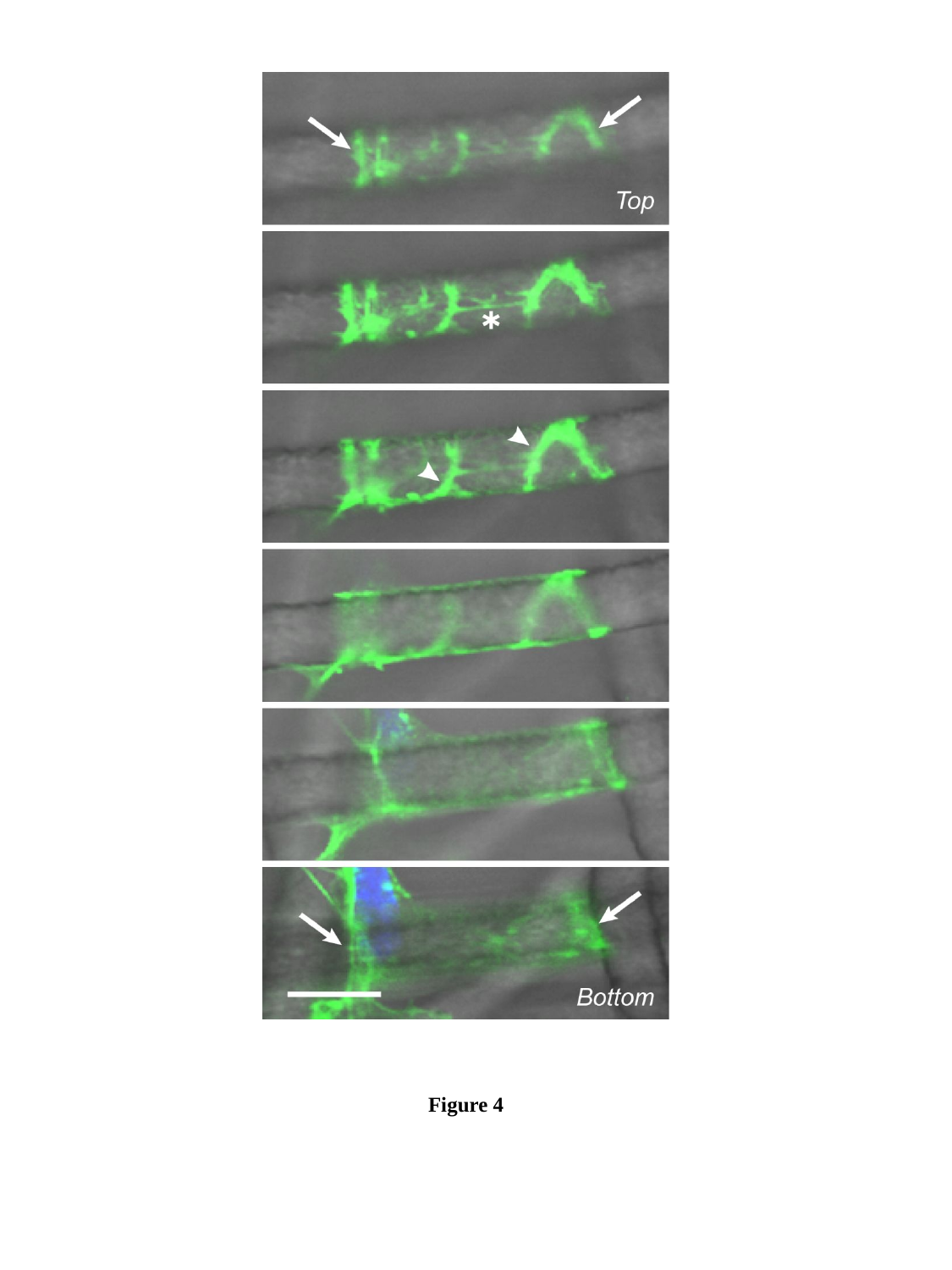

Figure 4

## Slide 5
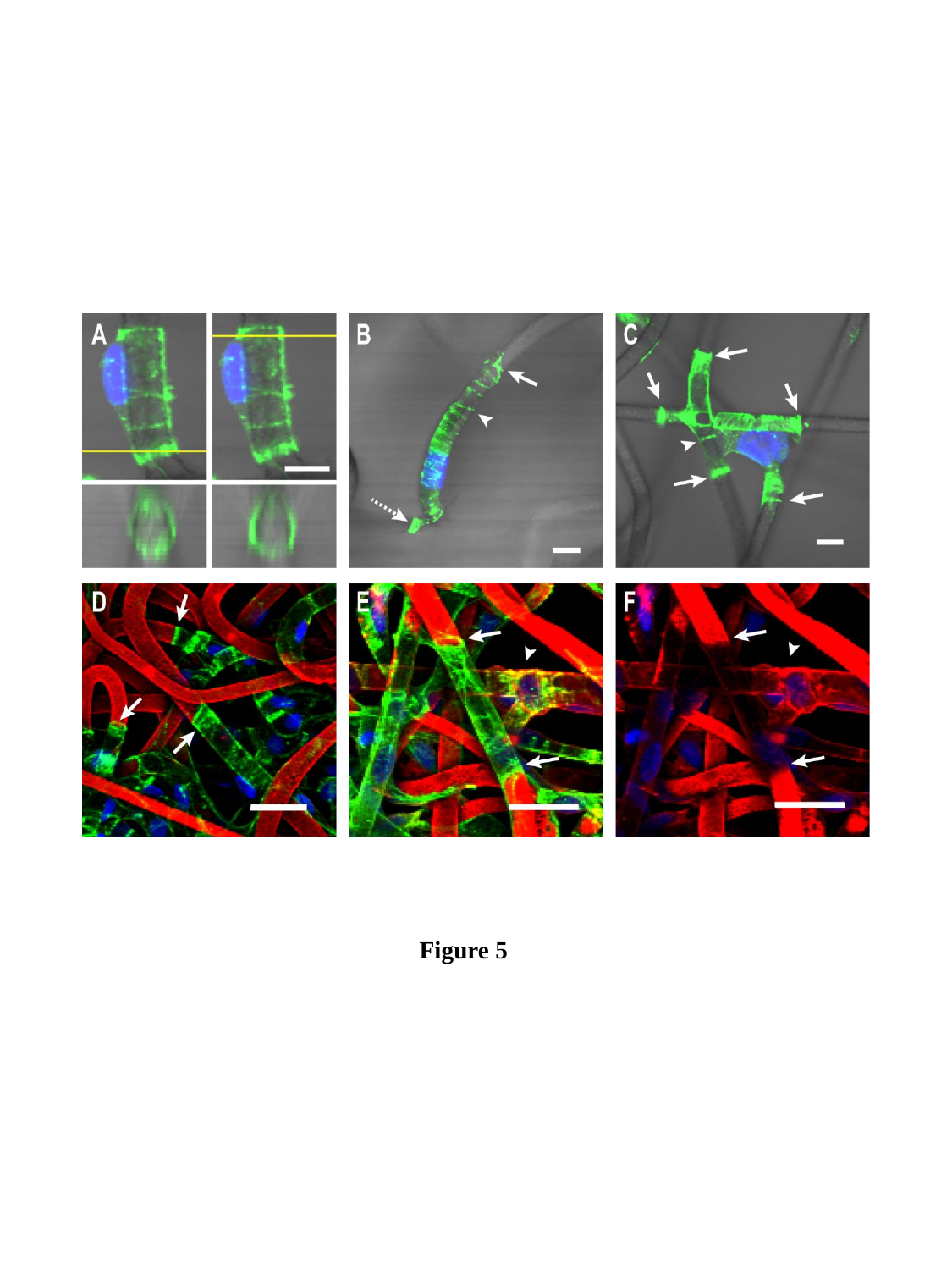

Figure 5

## Slide 6
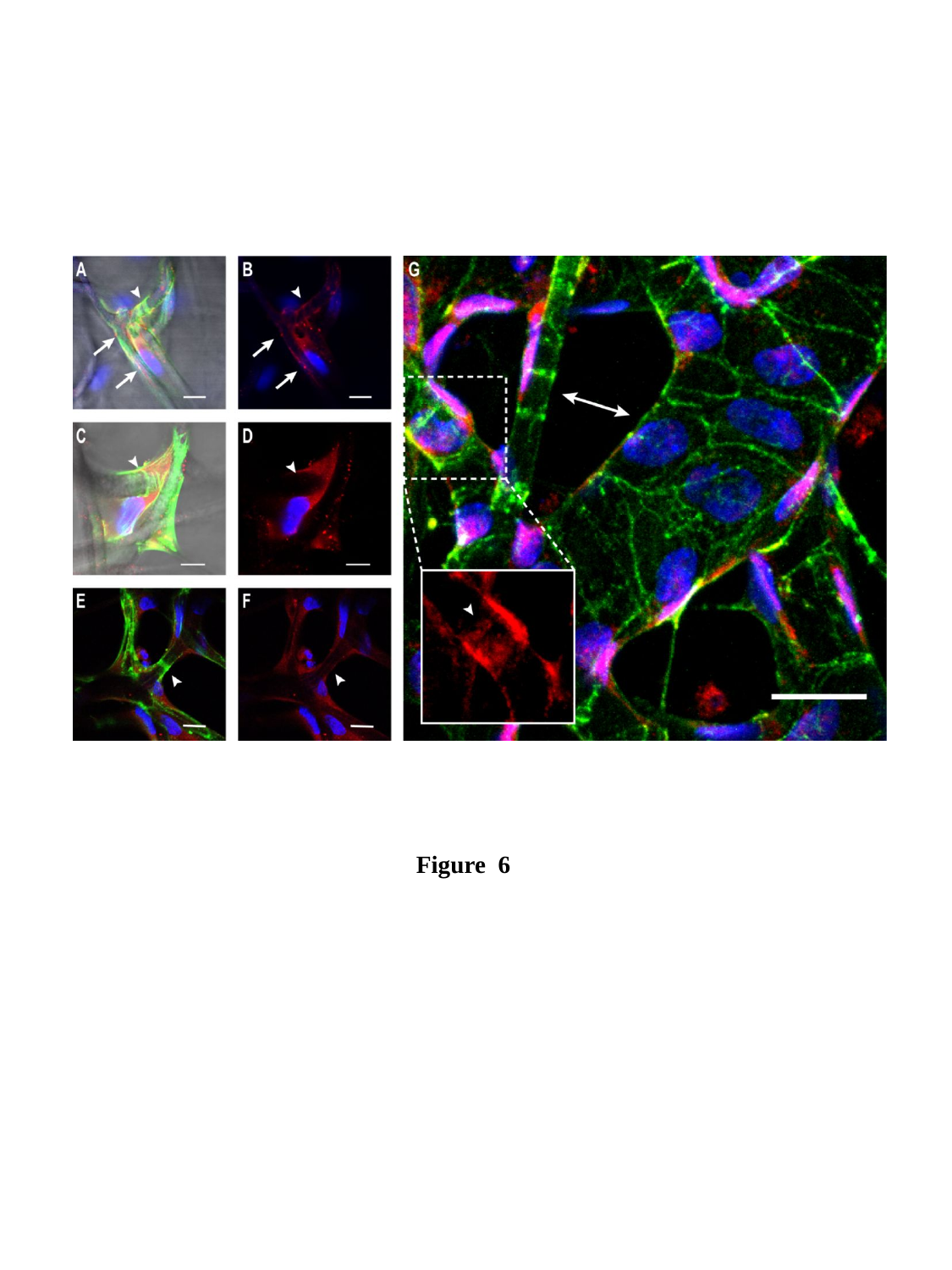

Figure 6

## Slide 7
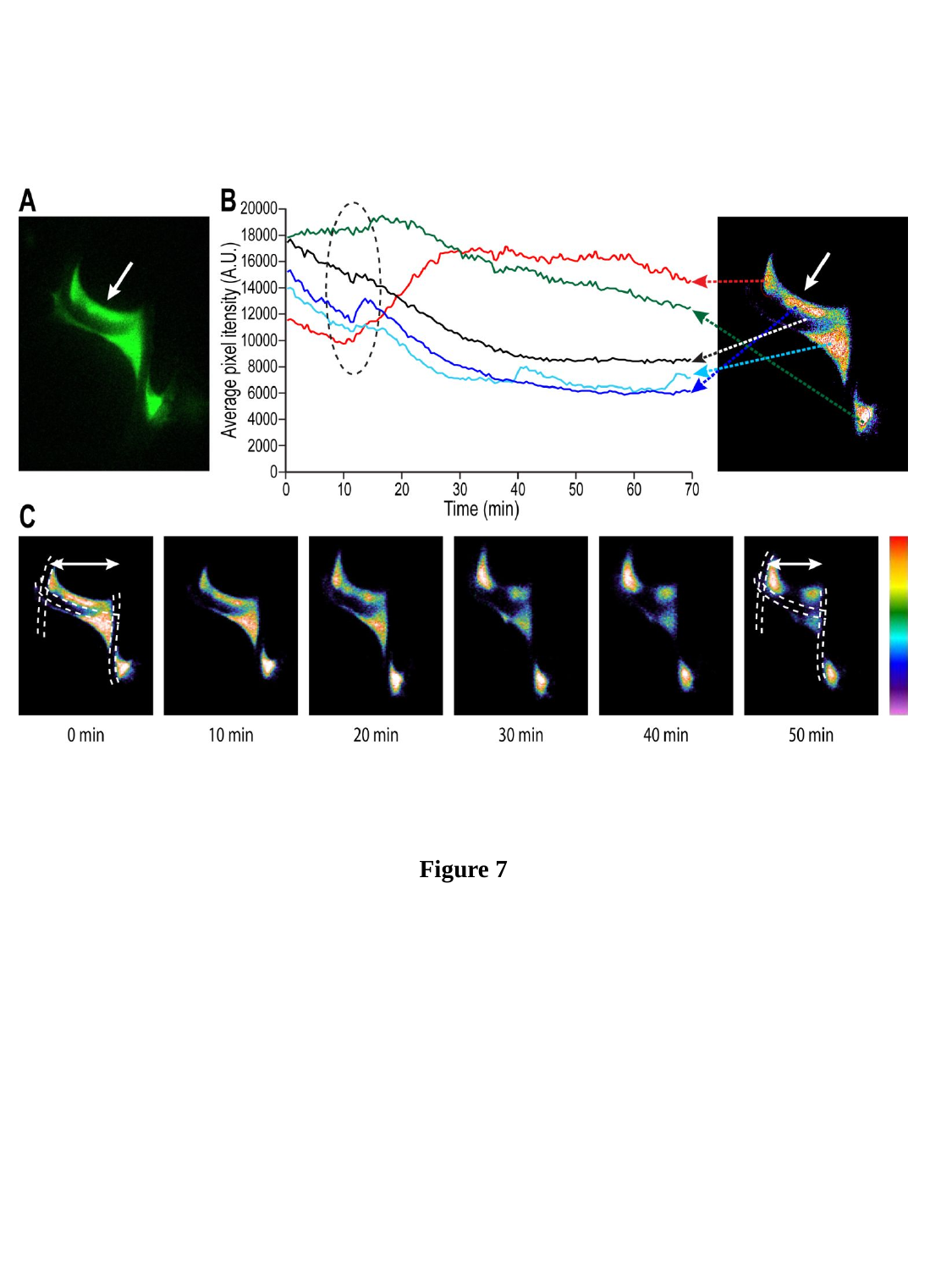

Figure 7

## Slide 8
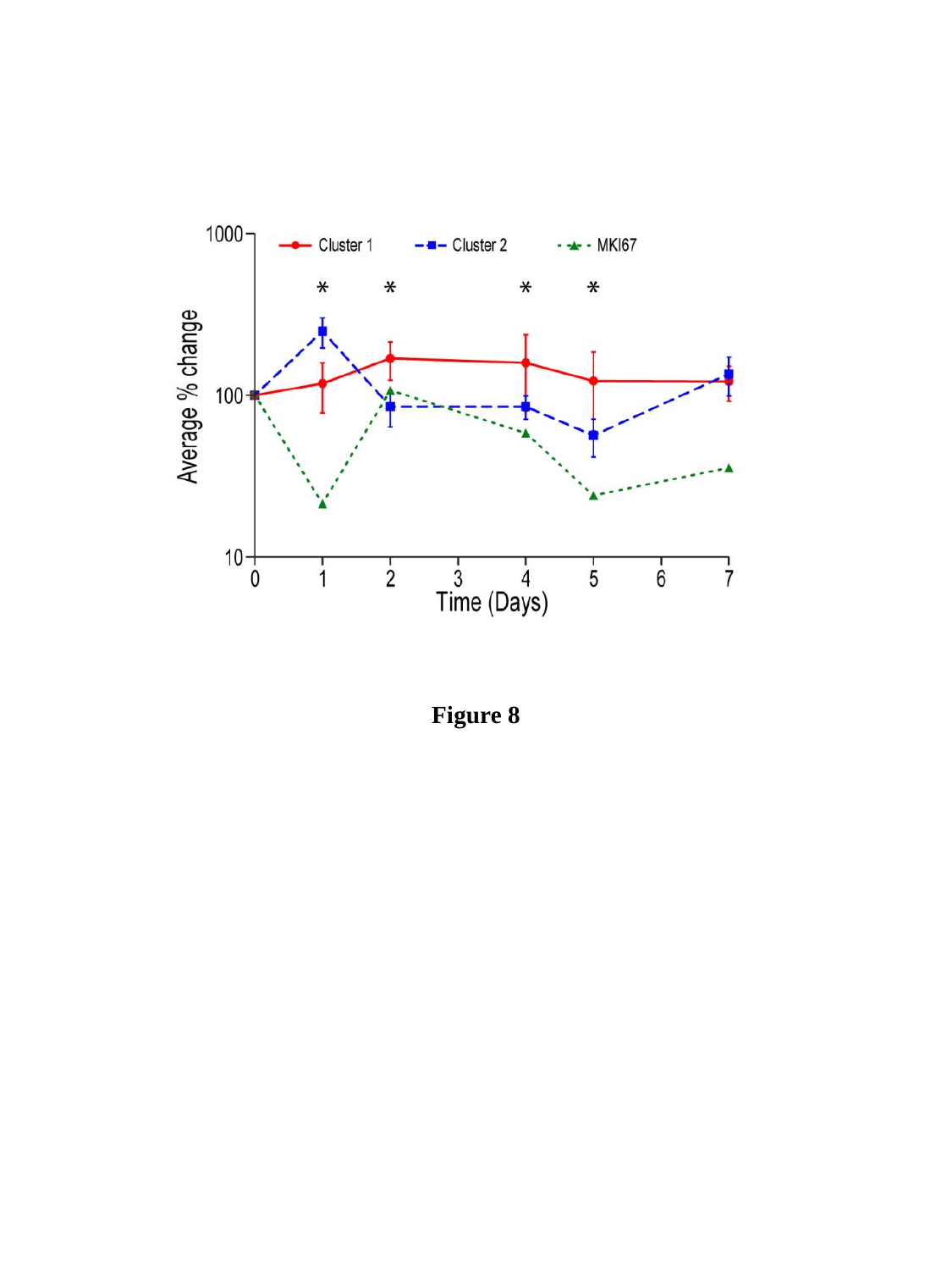

Figure 8
