# Supplementary material for: Actin Grips: Circular Actin-Rich Cytoskeletal Structures that Mediate the Wrapping of Polymeric Microfibers by Endothelial Cells
Source: Biomaterials. Author manuscript; Available in PMC 2016 Jun 1. (PMC4418805; doi:10.1016/j.biomaterials.2015.02.034)
Supplement: 3 [file NIHMS662697-supplement-3.docx]

**Title:**

*Actin Grips: Circular Actin-Rich Cytoskeletal Structures that Mediate the Wrapping of Polymeric Microfibers by Endothelial Cells*

**Author Affiliations:**

Desiree Jones*^a^*, DoYoung Park*^b^*, Mirela Anghelina*^a^*, Thierry Pecot*^a^*^,b^, Raghu Machiraju*^b^*, Ruipeng Xue*^c^*, John Lannutti*^c^*, Sara Cole*^d^*, Leni Moldovan*^a^*, and Nicanor I. Moldovan*^a*^*

Departments of *^a^*Internal Medicine, *^b^*Computer Sciences and Engineering, *^c^*Materials Sciences, and *^d^*Campus Microscopy and Imaging Facility, The Ohio State University, Columbus, OH

**Corresponding Author:**

Nicanor I. Moldovan, Ph.D., Department of Internal Medicine, Division of Cardiovascular Medicine, 460 W. 12 Ave, Room 306, Biomedical Research Tower

The Ohio State University, Columbus, OH 43210

Email: mnmoldova@yahoo.com


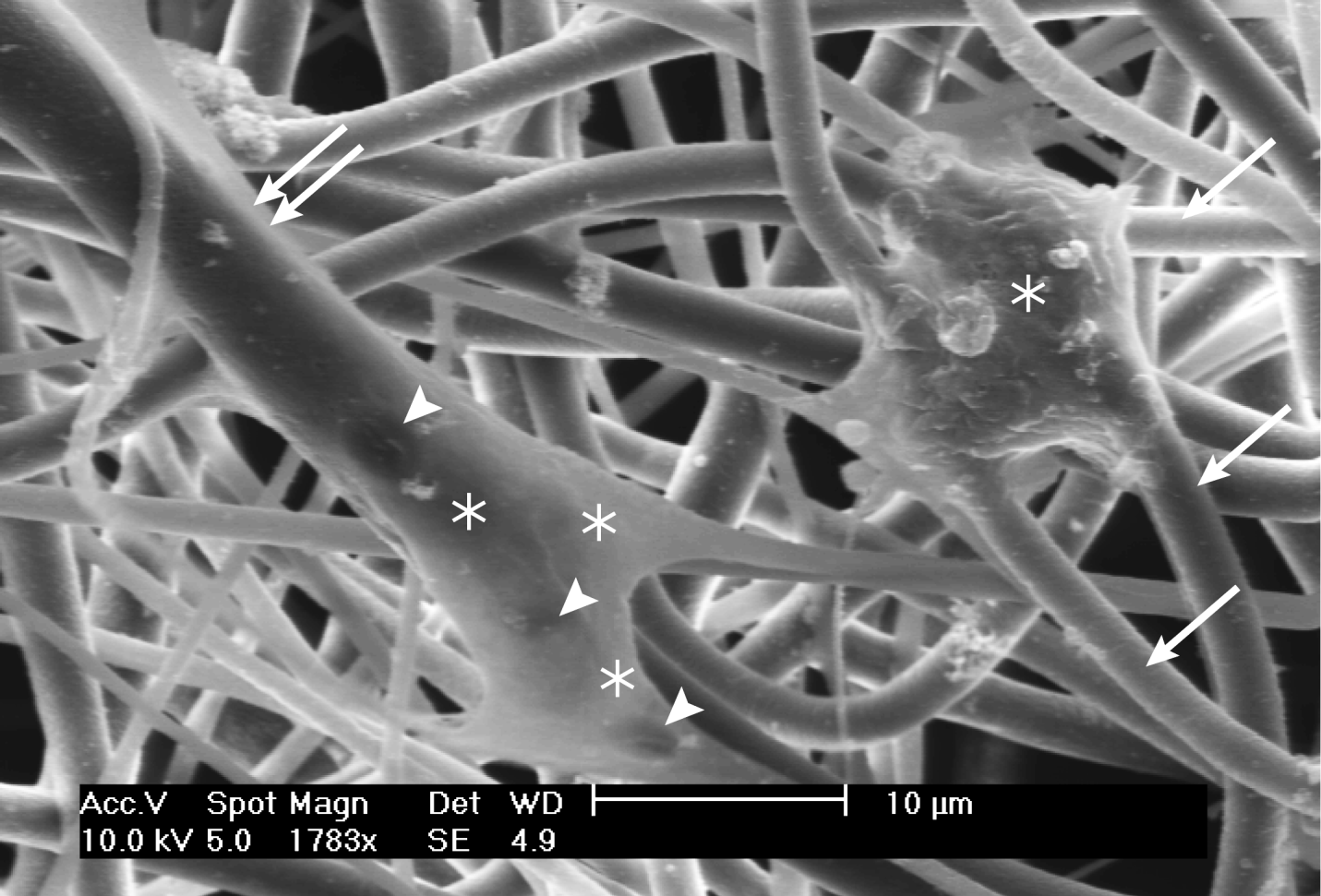


**Figure S1. Scanning electron microscopy (SEM) of a PCL scaffold and of attached ECs**. The image shows that the scaffold-attached ECFCs could engage either a single larger fiber (double-arrow), or several smaller fibers (arrows). In the first instance the cells became very attenuated (stars), including their nuclei, still visible in relief (arrowheads).


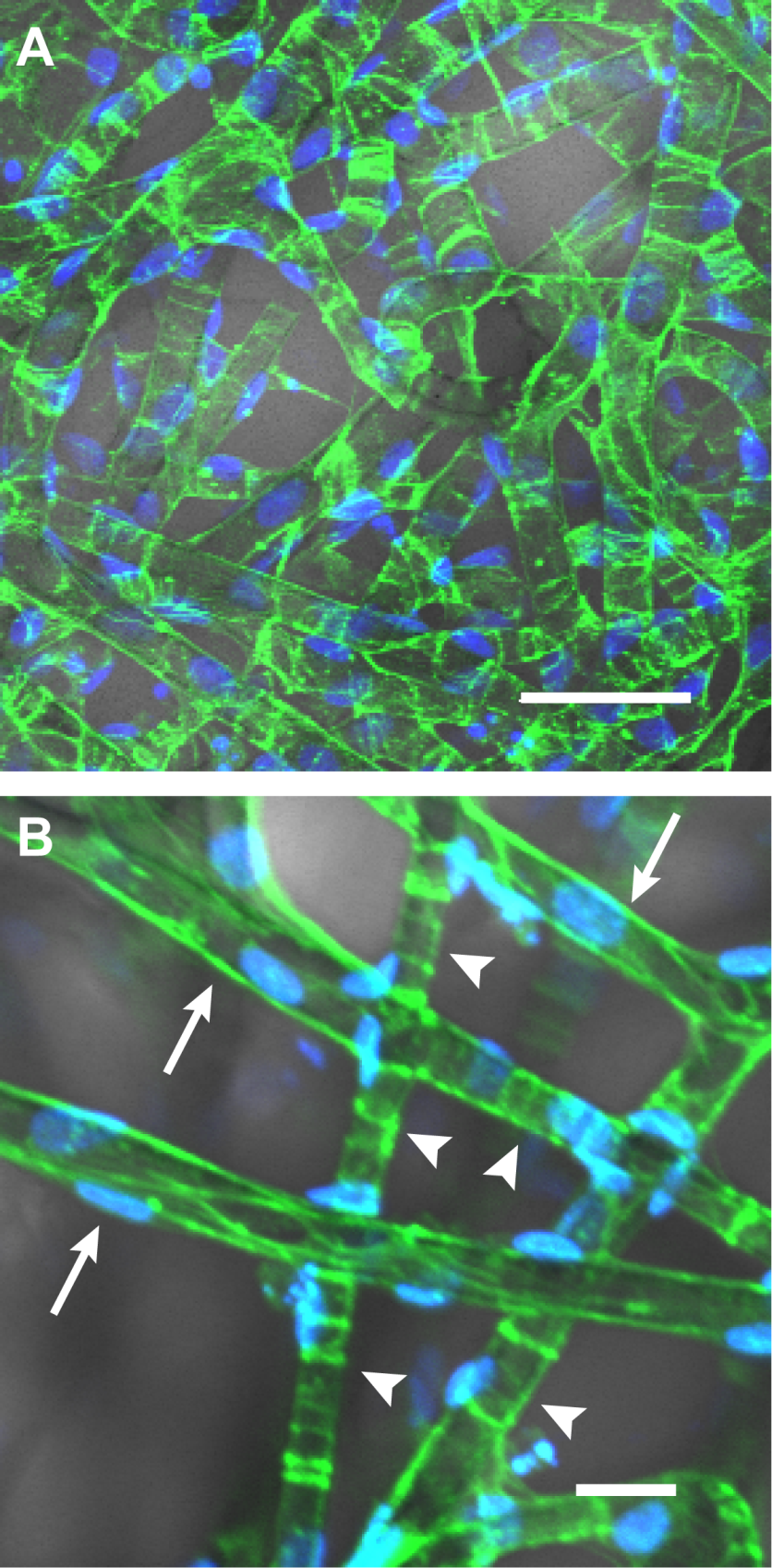


**Figure S2. Extent of PCL scaffold covering with HUVECs. A.** SMFs covering after 10 days of incubation; note that almost all fibers were wrapped by AGs-containing cells. **B.** Co-existence in cells attached to SMFs of comparable diameter of stress fiber-like actin filaments placed longitudinally vs. the scaffold (arrows), with transversally oriented AGs (arrowheads). **A, B,** overlay of DIC and fluorescence microscopy; two-dimensional projections of confocal z-stacks (green = F-actin, blue = DAPI). Scale bars: A: 50 μm; B: 20 μm.


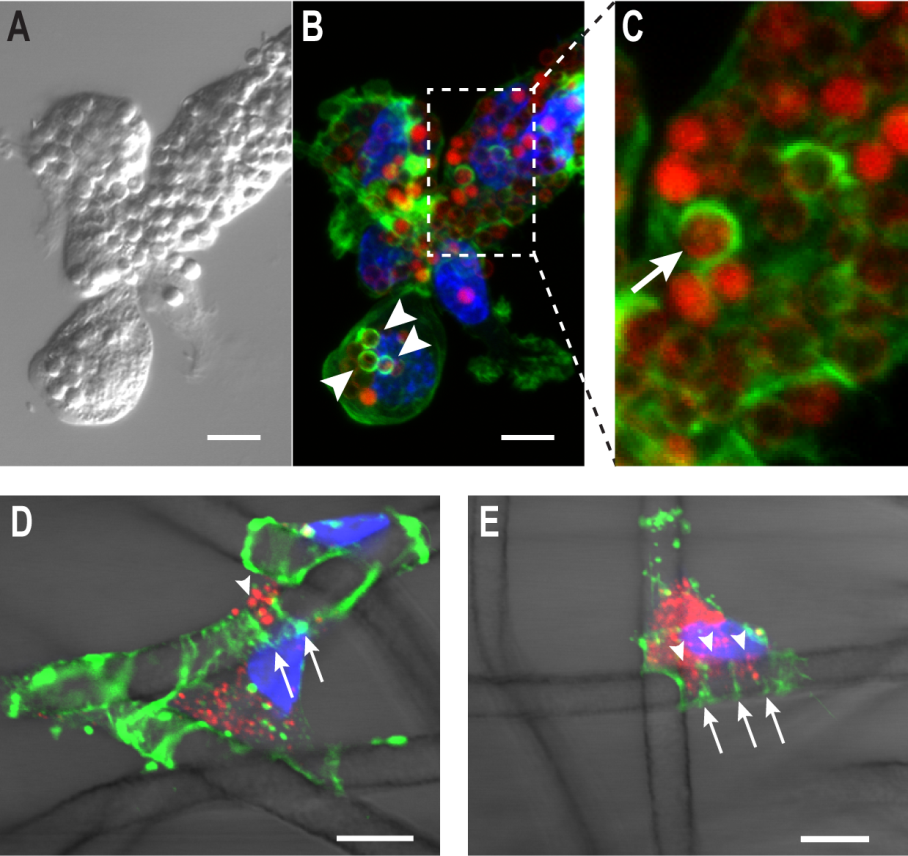


**Figure S3. Relationship between AGs and phagocytic activity in HUVECs. A-C.** *Bona fide* phagocytosis in HUVEC of antibody-covered polystyrene beads, providing a positive control for cortical actin organization around phagosomes in this cell type (**B,** arrowheads).The images represent phase contrast and fluorescence microscopy of HUVECs incubated with beads in suspension, followed by fixation, permeabilization, and staining. **C.** Higher magnification of **B**, showing the formation of an U-shaped ‘actin cup’ (arrow), characteristic for phagocytic internalization, around a more superficial bead in the process of engulfment. **D**. Optical sectioning through two SMFs-attached cells (F-actin, green) showing internalized PKH26 particles (arrowhead, red), in spaces limited by AGs (arrows). No F-actin organization is detectable in contact with the SMFs within these regions, arguing that AGs are not directly involved in phagocytosis. **E**. AGs (arrows) alternating with three phagosomes (arrowheads) within a fiber-attached cell. Note that AGs in this cell contained fewer microfilaments, and displayed a conspicuous beaded appearance. Images are two dimensional projections of confocal z-stacks. SMFs are visualized by DIC. F-actin (green), beads (red), and nuclei (blue) **B**-**C** are two-dimensional projections of confocal z-stacks. Scale bars: 10 μm.


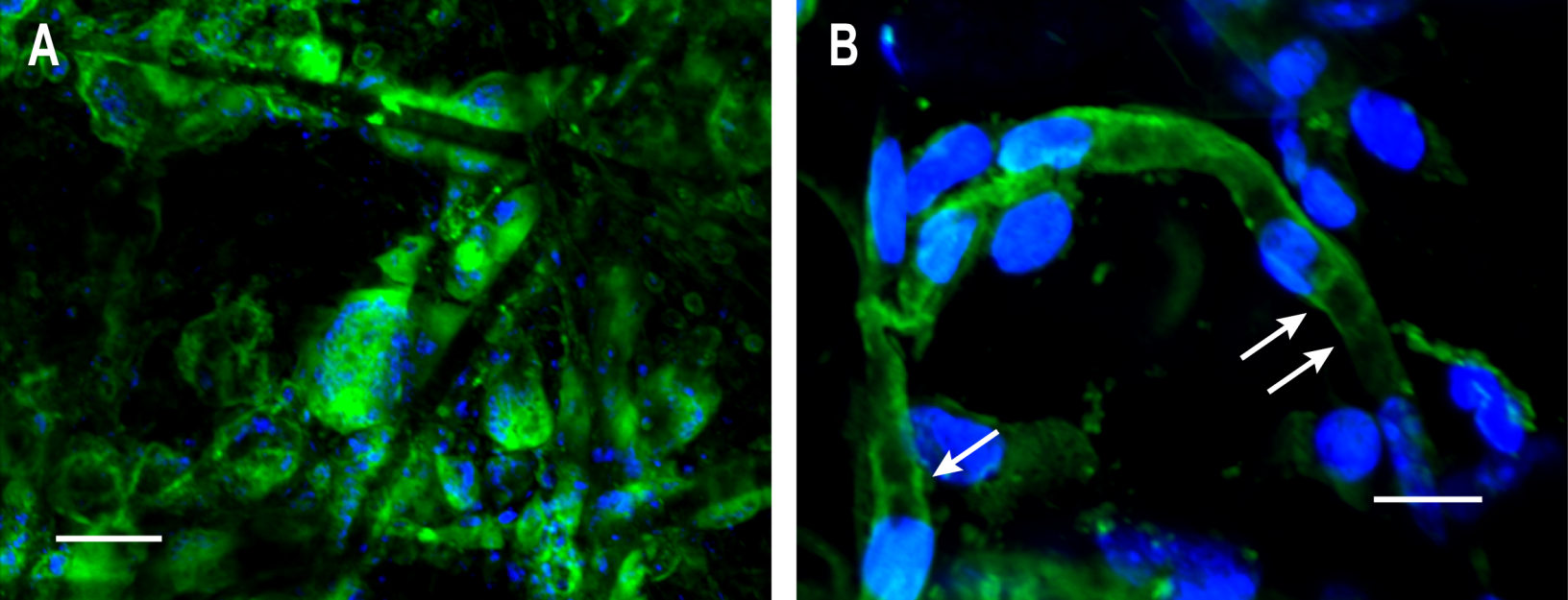


**Figure S4. Pattern of F-actin in cells with phagocytic capabilities *in vivo*. A**. F-actin in was found in a patchy distribution, without relationship with the SMFs, in macrophages and giant cells attached to a PCL scaffold retrieved after 6 weeks from a subcutaneously implanted mouse. **B.** In contrast, F-actin presented an occasionally transversal distribution to the long axis of the cell in neo-vascular capillaries growing in Matrigel plugs implanted subcutaneously in mice for 6 weeks,. Magnification bars, 30 µm.


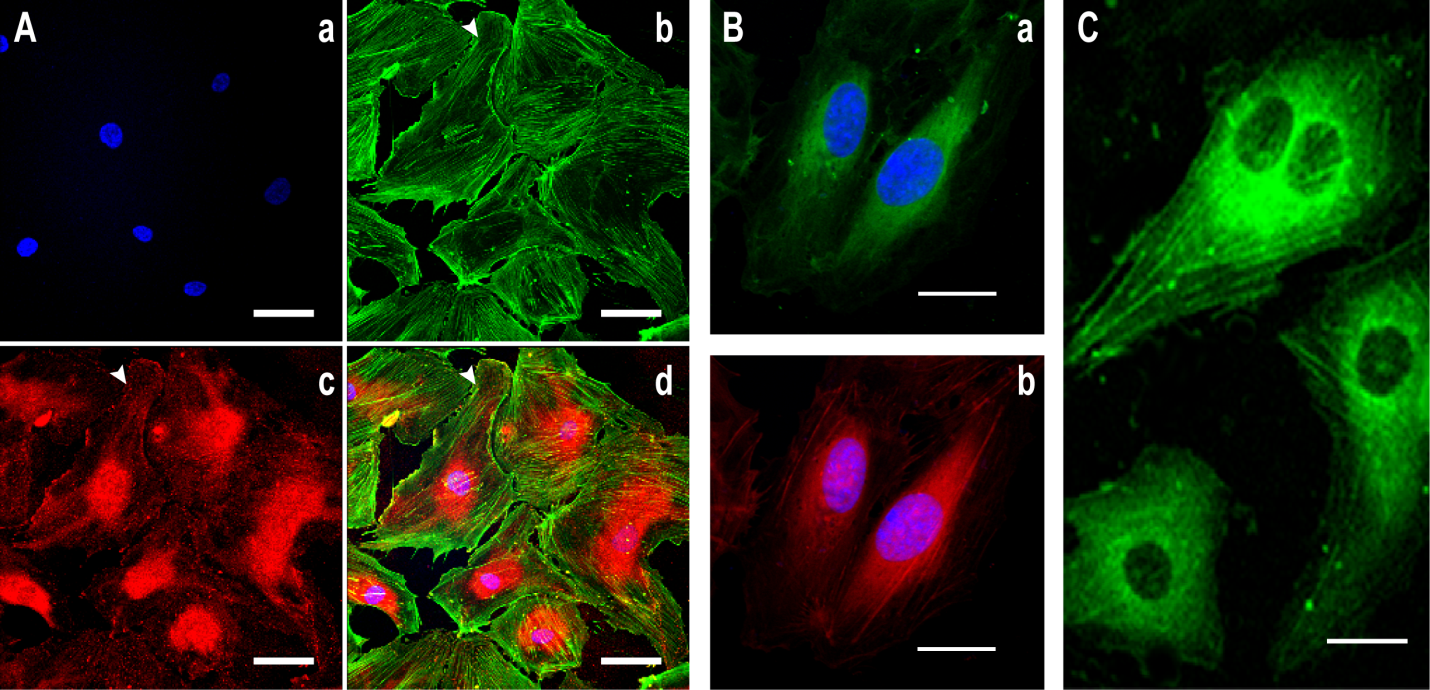


**Figure S5.** **Detection of cytoskeletal proteins in ECs in two-dimensional cultures. A.** Immuno-staining for paxillin (red) in TCPS-attached HUVECs (staining control for Fig. 6A,B). Note the localization of FAs as concentrated staining at the ends of stress fibers, detected by the fluorescent phalloidin (**b-d**, arrowheads). **B**. Co-localization of F-actin (identified with fluorescent phalloidin, green) with the fine network of intermediate filaments containing vimentin (red) (staining control for Fig. 6E,F). **C.** Incorporation of GFP-actin in stress fibers and marginal ruffles in 2D-cultured HUVECs (control for Fig. 7). **A-C** scale bars: 50 μm.
